# Comparison between Different Visual Acuity Tests and Validation of a Digital Device

**DOI:** 10.3390/vision8030057

**Published:** 2024-09-23

**Authors:** Blanca Montori, Teresa Pérez Roche, Maria Vilella, Estela López, Adrián Alejandre, Xian Pan, Marta Ortín, Marta Lacort, Victoria Pueyo

**Affiliations:** 1 Department of Pharmacology and Physiology, University of Zaragoza, Domingo Miral, s/n., 50009 Zaragoza, Spain; blancamontori97@gmail.com (B.M.);; 2 Aragón Health Research Institute (IIS Aragón), 50009 Zaragoza, Spain; 3 Ophthalmology Department, Miguel Servet University Hospital, 50009 Zaragoza, Spain; 4 Dive Medical S.L., Edificio Ceminem Spinup, Calle Mariano Esquillor Gómez, 50018 Zaragoza, Spain

**Keywords:** digital devices, visual acuity, visual testing, validation, HOTV, ETDRS, LEA symbols

## Abstract

Purpose: To compare different visual acuity (VA) tests (printed and digital, symbols and letters) and to validate a new device for VA testing called DIVE (Devices for an Integral Visual Examination). Methods: VA was tested in a wide spectrum of adult people with printed tests (ETDRS and LEA Symbols) and with two implemented tests in DIVE (HOTV and DIVE Symbols). We measured agreement between the different VA tests using the intraclass correlation coefficient and Bland–Altman method. In addition, we measured the repeatability of all tests. Results: Right eyes from 51 adult participants were included in the study. Correlation between tests was high (ICC from 0.95 to 0.97). Bland–Altman analysis showed good agreement among the different tests, with differences within reasonable clinical limits. However, slightly better VA values were obtained with DIVE HOTV and ETDRS, followed by LEA and DIVE Symbols. ETDRS had the best repeatability. Conclusion: The four evaluated VA tests provide comparable outcomes. In an adult sample, letter optotypes obtained better VA values than symbol optotypes. DIVE VA tests are reliable and well-correlated with printed VA tests.

## 1. Introduction

Eye disorders may require ongoing assessment of the visual function for optimal management. Visual acuity (VA) is the most common measure of visual function and is broadly used for clinical and research purposes. In addition, many vision-screening methods are based on VA [1,2,3]. Therefore, it is important to assess VA with reliable and precise methods, and any new VA test has to be validated with gold standards before using it in clinical practice.

Currently, there are numerous visual tests that use different stimuli, methods of presentation or measuring scales. The design of the stimuli is crucial, since the patient has to recognize a concrete shape. There are charts based on letters, numbers, and symbols. The latter two are mostly used with children and require easy recognition of the symbols, such as the ones used in LEA optotypes [4,5]. The most frequent charts in clinical practice for the measurement of VA are based on letters, such as ETDRS (Early Treatment Diabetic Retinopathy Study), HOTV, or Snellen.

Another source of variability is the algorithm of presentation of the stimuli and the conditions to run them. There are tests implemented in back-lighted boxes, on printed charts, via projector scopes, or displayed on computer screens_._ Factors such as luminance, contrast, exposure time, or color can affect VA outcomes [6,7], and sometimes, tests lack calibration or regular checking of these factors.

Recent technological advances are a valuable opportunity to standardize visual testing, allowing better control of stimuli presentation or data collection. However, as part of the development of any new diagnostic test, it is necessary to compare its performance with existing gold-standards. Although finding null differences between two tests is extremely rare, agreement analysis can confirm that differences are small enough to be considered clinically insignificant and, therefore, acceptable for clinical practice.

The aim of the present study was to compare different VA tests (printed charts and digital optotypes, based either on letters or symbols) and to validate VA tests implemented in a new digital device called DIVE (Device for an Integral Visual Examination).

## 2. Material and Methods

### 2.1. Study Participants

The study included participants recruited from the Pediatric Ophthalmology Unit (employees and relatives of the patients) of the Miguel Servet University Hospital from August 2022 to October 2022. As a validation study, the criteria were wide. The only requirement was related to the participant’s age, selecting only cooperative adults, to avoid lack of attention as a source of bias. All the participants provided informed consent.

#### 2.1.1. Inclusion Criteria

- Able to understand and comply with the testing protocol.
- Age from 15 to 68 years.

#### 2.1.2. Exclusion Criteria

- Not consenting to participate in the study.
- Bad general health state which does not allow a correct examination.
- Recent ocular surgery or ocular problems in their right eye.

### 2.2. Protocol

VA was measured using four different tests: two printed tests, LEA Symbols and ETDRS charts (Precision Vision, Inc., La Salle, IL, USA), and two digital tests implemented in DIVE (DIVE Medical S.L., Zaragoza, Spain), DIVE Symbols and DIVE HOTV. All four tests were performed twice on the same day by the same optometrist. The order of the examinations was randomly assigned at the inclusion in the study.

The testing procedure involved positioning patients on a chair 3 m away from the stimuli. The procedure was conducted monocularly and without optical correction, so to ensure a wide range of VA values. Only the right eye of every patient was tested and included in the statistical analysis. Before testing, every patient had an automatic refraction, and we measured their optical correction.

Response time was not limited, and participants were encouraged to fulfill the test but without urging. All the tests were time measured. Answers were provided aloud, and the optometrist noted the result.

### 2.3. Printed Charts (LEA Symbols and ETDRS)

The printed charts, LEA and ETDRS, use black figures against a white background. These two tests were conducted under photopic conditions.

The LEA Symbols chart uses four different recognizable shapes (house, apple, circle, and square). The chart for 3 m testing (version #250220) combines 5 figures in each line.

The ETDRS chart utilizes combinations of Sloan letters in different versions. For the present study, ETDRS charts 1 and R were used. VA values were recorded and adapted to a 3 m distance.

For the ETDRS and LEA Symbols, the size of the symbols is equal to the space between them. The range of the optotypes is from 1 to −0.4 LogMAR, and the steps for testing are 0.1 LogMAR.

The LEA Symbols and ETDRS printed tests were installed on a mobile trolley, externally and indirectly illuminated.

As a usual procedure in eye clinics, the tests began with the right eye. People were instructed to read all the stimuli they were able to and they were stopped when reading three incorrect letters or symbols in a row (line assignment method). VA value corresponded to the last line correctly identified. VA scores were noted in a LogMAR scale.

### 2.4. DIVE Tests (DIVE Symbols and DIVE HOTV)

The digital tests (DIVE symbols and HOTV) were performed using a prototype of DIVE device (DIVE Medical SL, Spain). The system was made up of a tablet that has a 12-inch screen with a resolution of 2160 × 1440 pixels with 216 PPI. The distance from the screen to the observer’s eyes was set at 3 m. The screen was regularly calibrated with a Datacolor SpyderX calibrator (gamma 2.20, white dot 6500 K, and 120 cd/m^2^). The DIVE tests were completed with the light from the tablet as the only light source, without external lights.

**Figure 1:**
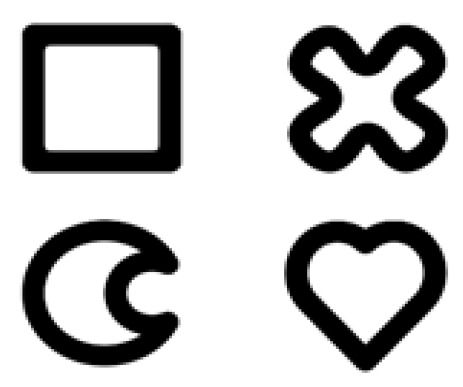
DIVE Symbols.

DIVE Symbols test includes four stimuli: a square, a heart, a moon, and a cross (Figure 1). They were selected after an iterative design process, in which eight different shapes were tested between adults and children. The first step for each shape was to be as recognizable as the LEA Symbols, at one determined size. The selected symbols were then checked to ensure that they were equally recognizable to each other. The trials were conducted among approximately 30 people.

Stimuli were presented one by one on the center of the screen against a plain white background. The figure was selected randomly among the four symbols, and it was surrounded by four lines (above, below, right, and left) to emulate the crowding effect.

DIVE Symbols were shown taking into account the following principles: The thickness of the lines within the stimulus determined the VA values. It was one-seventh of the stimulus height.

The HOTV test implemented in DIVE used the same principles: one letter on the center of the screen and bars around the letter for crowding effect as well.

The participant named the symbol aloud, and the optometrist recorded the answer as true or false on a keyboard. If it was correct, DIVE showed a smaller symbol or letter. A psychophysical procedure was followed to determine the steps in the stimuli size. The range of the stimuli displayed was from 1.1 to −0.2 LogMAR.

### 2.5. Statistical Analysis

Statistical analyses were carried out with the statistical software SPSS 21.0 (SPSS Inc., Chicago, IL, USA).

Mean, ranges, and standard deviation reported the VA outcomes. Agreement between the tests and repeatability were assessed using the intraclass correlation coefficient (ICC) and Bland–Altman method.

The ICC is defined as the ratio of the between-subject variance to the total variance, composed of the between-subject and within-subject variance. The ICC ranges from 0 (no agreement) to 1 (perfect agreement).

Bland–Altman limits of agreement (LOAs) are calculated as the mean of the differences +/−1.96 × standard deviation.

## 3. Results

The right eyes from 51 participants were finally included in the sample (12 men and 39 women). The mean age was 40.63 years (y), with a standard deviation of 13.05. The ages ranged from 23 y to 68 y. All patients completed the four VA tests. Of the 51 patients included, 33 had optical correction.

The VA outcomes are shown in Table 1. Although there were only small differences among the VA outcomes measured by the different tests, the highest VA values were obtained with the DIVE HOTV test implemented in DIVE, followed by the ETDRS, LEA, and DIVE Symbols.

The mean testing time was 34 s (s) with ETDRS, 43 s with LEA Symbols, 38 s with DIVE Symbols, and 32 s with DIVE HOTV. These differences were statistically significant (*p* = 0.003).

The ICC with the measure of absolute agreement showed good agreement in all the cases, with a range of values from 0.95 to 0.97 (Table 2).

**Figure 2:**
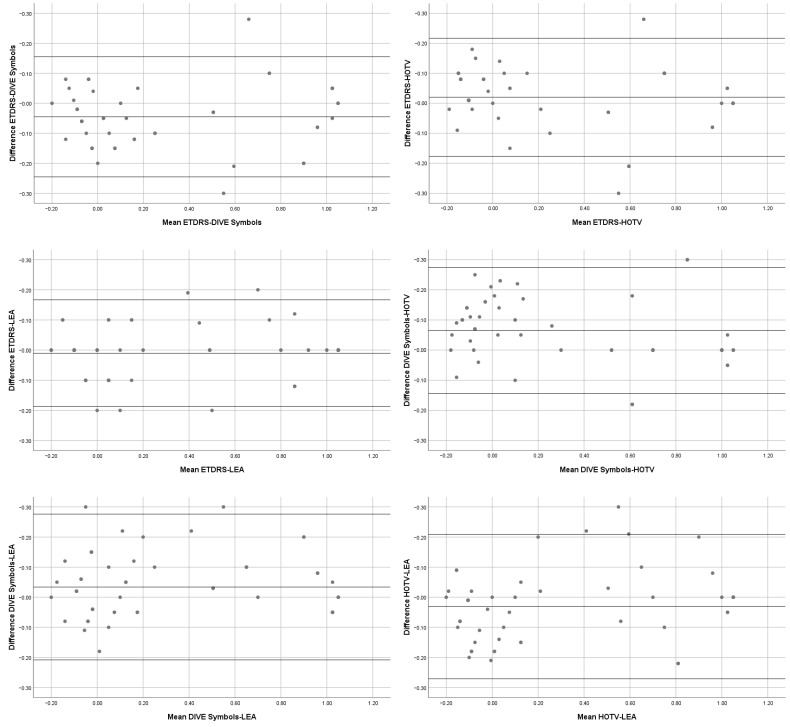
Bland–Altman graphics for tests comparison.

We used Bland–Altman plots to quantify the agreement among VA measurements. The Bland–Altman plots (Figure 2) compare the difference between two clinical outcomes (*y* axis) against the mean of these two values (*x* axis). The limits of agreement are exposed in Table 3.

We further divided the sample according to visual acuity levels: ETDRS ≥0.4 LogMAR (*n* = 18) and ETDRS < 0.4 (*n* = 33). The ICC results for every test were all between good and excellent agreement (0.77 to 0.93), with better values in the lower-visual-acuity group.

**Figure 3:**
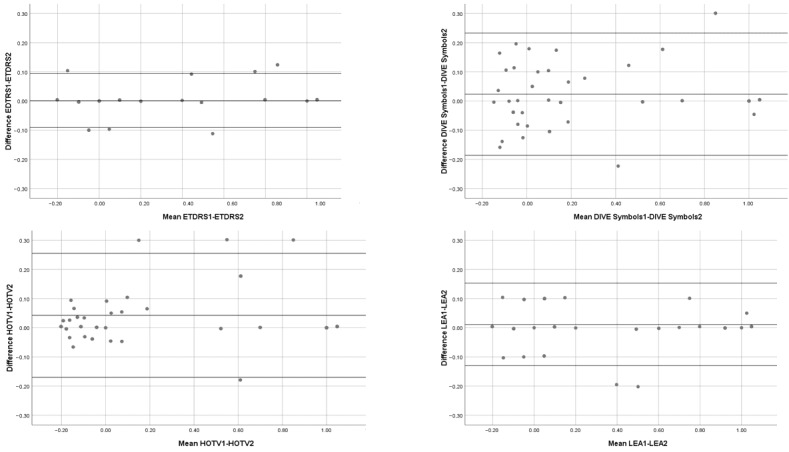
Repeatability of the tests.

We measured the repeatability of the tests with the ICC (Table 4) and the Bland–Altman method (Figure 3). The best repeatability was reached by ETDRS test.

## 4. Discussion and Conclusions

VA testing is essential to evaluate visual function. Its definition refers to the ability to recognize high-contrast stimuli with a subtending known angle [8]. According to these principles, different methods have been designed, taking into account the age and capacity of the patients.

For literate people, letters are a good method of recognition; HOTV, Snellen, and ETDRS are the most frequently used charts in clinical practice. The Snellen chart has a series of drawbacks, such as a different number of letters at each level and different vertical and horizontal distance between stimuli [9]. These limitations have been largely overcome with the LogMAR acuity charts, such as the ETDRS, which is considered the gold standard tool for the measurement of VA both in clinical practice and in research [10,11].

HOTV optotype is composed of the letters H, O, T, and V. All these letters are symmetric, which avoid the common mistake of inverting letters; therefore, it is of great value for testing children at the age of starting reading, around 4–5y [12,13].

Symbols are mainly used in children and patients who do not know the letters. LEA Symbols, tumbling E, and Landolt C are the most common ones; these last two, which represent visual resolution acuity, rely on the child’s spatial perception, and sometimes, this ability is not well developed until the age of 4y [14].

LEA Symbols was designed to avoid some cultural barriers using common pictures (square, circle, house, heart) and speech problems or shyness with the option of matching figures [5,15]. Many studies have compared LEA symbols and letter optotypes, most of them showing good agreement [16,17,18]. However, the question about comparison between symbols and letters is still open, with some works addressing a tendency to poorer VA values when measuring with LEA [19,20], while others show the contrary [21].

We compared letters and symbols in an adult sample to clarify the effect of the stimulus design on VA, avoiding the bias of attention in children. Our patients obtained slightly better results with letter charts than with symbols. LEA and DIVE Symbols were harder to recognize than the letters of ETDRS and DIVE HOTV. Differences could be due to familiarity with stimuli, with letters being more common in adulthood than symbols. DIVE Symbols obtained the lowest VA values. However, agreement between this test and the other three was good, as reported by the ICCs, and therefore, we can hypothesize a systematic bias of poorer VA with DIVE Symbols.

Photometric conditions vary across VA tests and can lead to a lack of standardization. In this sense, digital devices have more control of luminance conditions. Livingstone et al. reported better accomplishment of international photometric standards with iPad tablet devices than with retro illuminated ETDRS charts in a standard clinical use [22]. Higher contrast levels in digital devices have been pointed out as the reason for better visual acuities [23]. This could explain the slightly better VA values obtained by DIVE HOTV compared to ETDRS, being both letter optotypes. However, it should be noted that levels cannot be easily assessed in printed tests; therefore, direct comparisons cannot be performed.

Pixelization methods of digital screens appeared as a limitation to offer small visual stimuli and therefore to test high visual acuities. However, the availability of high resolution screens allows for displaying enough stimuli sizes to assess all ranges of VA, even in short distances to the viewer [24]_._ Actually, clinical studies have demonstrated no relevant effect of pixelization when comparing digital and printed charts [25].

Another source of bias in digital screens can be glare that results in significantly poorer VA values. Although this finding has been described in studies with tablets without anti-glare [25], in our experience, this factor was not present. It could be due to the relative control of the external light sources in a clinical setting.

DIVE used one isolated stimulus on the screen, surrounded by four bars. This method has been called contour interaction. It differs from the crowding effect that is the degradation of VA when a target is flanked by similar stimuli [26]. Crowding effect was present in printed charts as ETDRS and LEA Symbols, while in DIVE testing, there was only contour interaction, which seems to have less influence on VA than the design of the stimuli itself.

Since the arrival of portable digital technology, there have been an increasing number of studies that compare traditional versus digital visual tests. Most of them highlight the good correlation between gold standards and these emerging applications, displayed on tablets and smartphones [27,28,29,30,31]. In our study, the values obtained by the different tests substrate a reasonable agreement. The difference between the highest mean (with DIVE HOTV) and the lowest (with DIVE Symbols) was 0.15 LogMAR, which is clinically acceptable. DIVE tests demonstrated a good correlation with printed tests widely used, which make it a suitable option to measure VA.

Repeatability was excellent in all four tests according to ICC common interpretation [32], and ETDRS reached the best values. All tests but ETDRS had better VA values the second time the tests were performed. However, the learning effect was avoided in DIVE tests, as it uses random stimuli.

The small number of patients comprised may limit the generalizability of the study, specifically the absence of children. The line assignment method for ETDRS and LEA (VA as the last line at which the patient read 3 out of 5 letters or symbols) has poorer results in test–retest variability than the other methods (letter by letter or probit analysis), and this fact may affect the results of the study. Another source of variability could be the different conditions under which the tests were performed: higher external luminance levels for printed tests and DIVE tests performed with the monitor as the only light source could result in differences in contrast levels. However, the nature of the digital and printed tests makes this potential bias difficult to avoid.

Finally, the use of optical correction could lead to different results, but the current perspective makes the conclusions more generalizable.

The four VA tests compared in this study provide good agreement between them, with differences small enough to be considered acceptable for clinical purposes. VA tests based on letters showed slightly better values. VA tests implemented in DIVE, both based on letters and on symbols, are comparable to existing gold standards, confirming the validity of the device.

## Figures and Tables

**Table 1:** VA values measured by the four different tests. LogMAR.

	N	Minimum	Maximum	Mean	Standard Deviation
DIVE HOTV	51	−0.2	1.1	0.26	0.46
ETDRS	51	−0.2	1.0	0.28	0.44
LEA Symbols	51	−0.2	1.1	0.29	0.43
DIVE Symbols	51	−0.2	1.0	0.33	0.43

**Table 2:** Intraclass correlation coefficient (ICC) between the tests.

	ICC	*p*
ETDRS vs. DIVE Symbols	0.96	<0.001
ETDRS vs. DIVE HOTV	0.97	<0.001
ETDRS vs. LEA Symbols	0.97	<0.001
DIVE Symbols vs. DIVE HOTV	0.96	<0.001
DIVE Symbols vs. LEA Symbols	0.95	<0.001
DIVE HOTV vs. LEA Symbols	0.96	<0.001

**Table 3:** Bland–Altman limits of agreement between the tests.

	Mean of Differences	Upper Limit of Agreement	Lower Limit of Agreement
ETDRS–DIVE Symbols	−0.045	0.155	−0.245
ETDRS–DIVE HOTV	0.020	0.217	−0.177
ETDRS–LEA Symbols	−0.010	0.167	−0.187
DIVE Symbols–DIVE HOTV	0.065	0.274	−0.144
DIVE Symbols–LEA Symbols	0.034	0.276	−0.208
LEA Symbols–DIVE HOTV	−0.031	0.209	−0.271

**Table 4:** ICC for repeated measures.

	ICC	*p*
ETDRS1–ETDRS 2	0.98	<0.001
DIVE Symbols 1–DIVE Symbols 2	0.91	<0.001
DIVE HOTV 1–DIVE HOTV 2	0.97	<0.001
LEA Symbols 1–LEA Symbols 2	0.95	<0.001

## Data Availability

The datasets used and/or analyzed during the current study are available from the corresponding author on reasonable request.

## References

[1] Evans J.R., Morjaria P., Powell C. (2018). Vision screening for correctable visual acuity deficits in school-age children and adolescents. Cochrane Database Syst. Rev..

[2] Clarke E.L., Evans J.R., Smeeth L. (2018). Community screening for visual impairment in older people. Cochrane Database Syst. Rev..

[3] Leibowitz H.M., Krueger D.E., Maunder L.R., Milton R.C., Kini M.M., Kahn H.A., Nickerson R.J., Pool J., Colton T.L., Ganley J.P. (1980). The Framingham Eye Study monograph: An ophthalmological and epidemiological study of cataract, glaucoma, diabetic retinopathy, macular degeneration, and visual acuity in a general population of 2631 adults, 1973–1975. Surv. Ophthalmol..

[4] Hered R.W., Murphy S., Clancy M. (1997). Comparison of the HOTV and Lea Symbols charts for preschool vision screening. J. Pediatr. Ophthalmol. Strabismus.

[5] Becker R., Hübsch S., Gräf M.H., Kaufmann H. (2002). Examination of young children with Lea symbols. Br. J. Ophthalmol..

[6] Johnson C.A., Casson E.J. (1995). Effects of luminance, contrast, and blur on visual acuity. Optom. Vis. Sci..

[7] Santucci G., Menu J.P., Valot C. (1982). Visual acuity in color contrast on cathode ray tubes: Role of luminance, hue, and saturation contrasts. Aviat. Space Environ. Med..

[8] Kniestedt C., Stamper R.L. (2003). Visual acuity and its measurement. Ophthalmol. Clin. N. Am..

[9] León Álvarez A., Estrada Álvarez J.M. (2011). Reproducibilidad y Concordancia Para la Carta Snellen y Lea en la Valoración de la Agudeza Visual en Infantes de Primaria. Investig. Andin..

[10] Bailey I.L., Lovie-Kitchin J.E. (2013). Visual acuity testing. From the laboratory to the clinic. Vision Res..

[11] Bokinni Y., Shah N., Maguire O., Laidlaw D.A.H. (2015). Performance of a computerised visual acuity measurement device in subjects with age-related macular degeneration: Comparison with gold standard ETDRS chart measurements. Eye.

[12] Simons K. (1983). Visual acuity norms in young children. Surv. Ophthalmol..

[13] Pan Y., Tarczy-Hornoch K., Cotter S., Wen G., Borchert M.S., Azen S.P., Varma R., Multi-Ethnic Pediatric Eye Disease Study (MEPEDS) Group (2009). Visual Acuity Norms in Preschool Children: The Multi-Ethnic Pediatric Eye Disease Study. Optom. Vis. Sci..

[14] Engin Ö., Despriet D.D.G., Van Der Meulen-Schot H.M., Romers A., Slot X., Sang M.T.F., Fronius M., Kelderman H., Simonsz H.J. (2014). Comparison of optotypes of Amsterdam Picture Chart with those of Tumbling-E, LEA symbols, ETDRS, and Landolt-C in non-amblyopic and amblyopic patients. Graefes Arch. Clin. Exp. Ophthalmol..

[15] Vivekanand U., Gonsalves S., Bhat S.S. (2019). Is LEA symbol better compared to Snellen chart for visual acuity assessment in preschool children?. Rom. J. Ophthalmol..

[16] Thomas J., Rajashekar B., Kamath A., Gogate P. (2020). Diagnostic accuracy and agreement between visual acuity charts for detecting significant refractive errors in preschoolers. Clin. Exp. Optom..

[17] Inal A., Ocak O.B., Aygit E.D., Yilmaz I., Inal B., Taskapili M., Gokyigit B. (2017). Comparison of visual acuity measurements via three different methods in preschool children: Lea symbols, crowded Lea symbols, Snellen E chart. Int. Ophthalmol..

[18] Cyert L. (2010). Effect of age using Lea Symbols or HOTV for preschool vision screening. Optom. Vis. Sci..

[19] Dobson V., Clifford-Donaldson C.E., Miller J.M., Garvey K.A., Harvey E.M. (2009). A comparison of Lea Symbol vs ETDRS letter distance visual acuity in a population of young children with a high prevalence of astigmatism. J. Am. Assoc. Pediatr. Ophthalmol. Strabismus.

[20] Dobson V., Maguire M., Orel-Bixler D., Quinn G., Ying G.-S., Vision in Preschoolers Study Group (2003). Visual acuity results in school-aged children and adults: Lea Symbols chart versus Bailey-Lovie chart. Optom. Vis. Sci..

[21] Ruttum M.S., Dahlgren M. (2006). Comparison of the HOTV and Lea symbols visual acuity tests in patients with amblyopia. J. Pediatr. Ophthalmol. Strabismus.

[22] Livingstone I.A.T., Tarbert C.M., Giardini M.E., Bastawrous A., Middleton D., Hamilton R. (2016). Photometric compliance of tablet screens and retro-illuminated acuity charts as visual acuity measurement devices. PLoS ONE.

[23] Tofigh S., Shortridge E., Elkeeb A., Godley B.F. (2015). Effectiveness of a smartphone application for testing near visual acuity. Eye.

[24] Aslam T.M., Parry N.R.A., Murray I.J., Salleh M., Col C.D., Mirza N., Czanner G., Tahir H.J. (2016). Development and testing of an automated computer tablet-based method for self-testing of high and low contrast near visual acuity in ophthalmic patients. Graefe’s Arch. Clin. Exp. Ophthalmol..

[25] Rosser D.A., Murdoch I.E., Fitzke F.W., Laidlaw D.A.H. (2003). Improving on ETDRS acuities: Design and results for a computerised thresholding device. Eye.

[26] Pluháček F., Musilová L., Bedell H.E., Siderov J. (2021). Number of flankers influences foveal crowding and contour interaction differently. Vis. Res..

[27] Bastawrous A., Rono H.K., Livingstone I.A.T., Weiss H.A., Jordan S., Kuper H., Burton M.J. (2015). The Development and Validation of a Smartphone Visual Acuity Test (Peek Acuity) for Clinical Practice and Community-Based Fieldwork. JAMA Ophthalmol..

[28] Rosenblatt A., Stolovitch C., Gomel N., BacharZipori A., Mezad-Koursh D. (2021). A novel device for assessment of amblyopic risk factors in preverbal and verbal children—A pilot study. Eye.

[29] Tiraset N., Poonyathalang A., Padungkiatsagul T., Deeyai M., Vichitkunakorn P., Vanikieti K. (2021). Comparison of Visual Acuity Measurement Using Three Methods: Standard ETDRS Chart, Near Chart and a Smartphone-Based Eye Chart Application. Clin. Ophthalmol..

[30] Rono H.K., Bastawrous A., Macleod D., Wanjala E., Di Tanna G.L., Weiss H.A., Burton M.J. (2018). Smartphone-based screening for visual impairment in Kenyan school children: A cluster randomised controlled trial. Lancet Glob. Health.

[31] Brady C.J., Eghrari A.O., Labrique A.B. (2015). Smartphone-Based Visual Acuity Measurement for Screening and Clinical Assessment. JAMA.

[32] Koo T.K., Li M.Y. (2016). A Guideline of Selecting and Reporting Intraclass Correlation Coefficients for Reliability Research. J. Chiropr. Med..

